# Type 2 Diabetes Mellitus, a Sequel of Untreated Childhood Onset Growth Hormone Deficiency Developing in a 17-Year-Old Patient

**DOI:** 10.1155/2018/4748750

**Published:** 2018-10-24

**Authors:** Rohan K. Henry, Ram K. Menon

**Affiliations:** ^1^Division of Endocrinology, Department of Pediatrics, Nationwide Children's Hospital, The Ohio State University College of Medicine, Columbus, OH 43205, USA; ^2^Division of Endocrinology, Department of Pediatrics, C.S. Mott Children's Hospital, Michigan Medicine, University of Michigan Medical School, Ann Arbor, MI 48109, USA

## Abstract

In a seminal report, a 17-year-old boy with panhypopituitarism had fatty liver (FL) amelioration with growth hormone (GH). By extension, since hepatic insulin resistance (IR) is key to FL and type 2 diabetes mellitus (T2DM), GH then may ameliorate the IR of T2DM. We present a 17-year-old nonobese female with untreated childhood onset growth hormone deficiency (CO-GHD) who developed type 2 diabetes mellitus (T2DM) and steatohepatitis with bridging fibrosis. Based on height z-score of – 3.1 and a history of radiation therapy as treatment for a medulloblastoma at 7 years of age, GHD was quite likely. GH therapy was, however, not initiated at 15 years of age (when growth was concerning) based on full skeletal maturity. After she developed T2DM, GHD was confirmed and GH was initiated. With its initiation, though insulin dose decreased from 2.9 (~155 units) to 1.9 units/kg/day (~ 100 units), her T2DM was, however, not fully reversed. This illustrates the natural history of untreated CO-GHD and shows that though hepatic IR can be ameliorated by GH, full reversal of T2DM may be prevented with irreversible hepatic changes (fibrosis). Clinicians caring for pediatric patients and otherwise should remember that, even in patients beyond the cessation of linear growth, GH can have a crucial role in both glucose and lipid metabolism.

## 1. Introduction

The role of growth hormone (GH) in growth promotion is well known by clinicians, however, less appreciated is its effect on metabolism in the well state. In 1936, Bernardo Houssay, M.D. in the New England Journal of Medicine, proposed that the anterior pituitary gland after the liver and pancreas plays a key role in glucose metabolism. That key role was later shown to be due in part to GH [1].

The effect of GH on glucose metabolism involves two phases: an initial phase which involves a decrease in glucose (an insulin-like effect) and a second phase which includes its effects on gluconeogenesis and fat mobilization [2]. In both states of growth hormone deficiency and excess, these effects on glucose metabolism will be altered.

Altered glucose and fat metabolism are important components of fatty liver (FL) and type 2 diabetes (T2DM), both states of hepatic insulin resistance (IR). In fact, a seminal case of fatty liver (FL) resolution with GH administration in a 17-year-old patient with panhypopituitarism treated initially with levothyroxine and hydrocortisone alone was reported by* Takano *et al. in 1997 [3]. This suggests that GH treatment of T2DM which may be associated with GHD has the potential to reduce the IR which may be present.

We outline a case of untreated childhood onset growth hormone deficiency (CO-GHD) who presented with type 2 diabetes mellitus (T2DM) and also steatohepatitis. We discuss her management and evidence from the basic sciences and clinical studies which show that her presentation with T2DM and steatohepatitis was likely associated with untreated GHD and that with GH supplementation her condition was ameliorated. Lastly, we discuss the implications of this case.

## 2. Case Presentation

**Figure 1:**
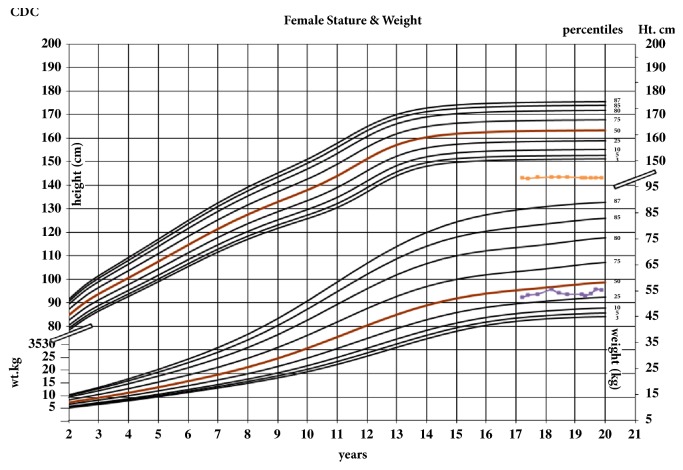
Patient's weight for age and height for age on CDC growth charts after being 17 years old.

A 17-year-old nonobese Caucasian female who had a history of a medulloblastoma diagnosed at 7 years of age was treated with radiation therapy. She subsequently developed TSH and GnRH deficiencies. Though GHD was suspected based on height (z-score of – 3.1; see Figure 1), treatment had not been initiated based on the initial management focus being to treat her medulloblastoma. At 15 years of age when her bone age showed full skeletal maturity, her parents were informed that GH therapy could not be pursued because her linear growth was complete.

On presentation, the patient's height was 141.3 cm (z= -3.1) and weight was 53 kgs (36^th^ percentile for age). Body mass index was 25.8 kg/m^−2^ (86^th^ percentile for age). Surveillance labs done at the oncology clinic showed glucosuria. Further testing showed HbA1c of 9.6% and on another day her fasting glucose was 277 mg/dL. Based on these results, diabetes mellitus was diagnosed.

When glutamic acid decarboxylase (GAD-65; Esoterix), islet-cell (Esoterix), insulin (Esoterix), and zinc transporter 8 (ARUP Laboratories) antibodies as well as DNA panel for maturity onset diabetes of youth (MODY) genes (HNF4*α*, GCK, IPF1, HNF1*α*, and HNF1*β*, [Athena Diagnostics]) returned all negative along with an elevated fasting C-peptide level of 3 ng/mL (normal: 0.4 - 2.1), T2DM was diagnosed. With the initiation of traditional basal/bolus insulin therapy using conventional dosing, a rapid escalation to peak total daily insulin dose of 2.9 units/kg/day (~ 155 units/day) was required to treat her refractory hyperglycemia. Treatment nonadherence was thought to be the unlikely cause of her increased insulin requirements based on the agreement between her insulin dosing and prescription refill data.

A comprehensive evaluation for conditions associated with IR was negative. However, based on Arginine/Clonidine stimulation testing showing peak GH level of 0.8 (normal: ≥ 10 ng/mL), a diagnosis of GHD was made. GH supplementation was initiated at 0.3 mgs daily and titrated based on IGF-1 levels.

After GH was started, her systolic and diastolic blood pressures (BP) which were mildly elevated between 124-136 and 77-89, respectively, became more normal. Despite this, lisinopril 5 mgs once daily was added for microalbuminuria.

With the diagnosis of T2DM and our patient having a significant family history of adverse cardiovascular risk factors, she was started on atorvastatin 10 mgs once daily. Within 2 months of therapy, her LDL cholesterol (LDL-C) decreased to 74 mg/dL.  Table 1 shows serial lipid profiles.

Though her diabetes was not fully reversed with GH, her HbA1c decreased to 5.9% and 5.8% at 6 and 19 months, respectively. Her insulin therapy requirement decreased to 1.9 units/kg/day (~ 100 units) at 12 months after the start of GH.

**Figure 2:**
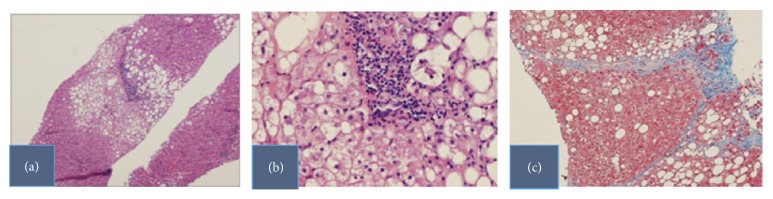
Slides from the patient's liver biopsy.** (a)** Low power liver section,** (b)** ballooning hepatocyte degeneration, a feature of hepatic cell death, and** (c)** intervening fibrous tissue seen as a terminal stage of liver injury. These all constitute steatohepatis with moderate steatosis.

Magnetic Resonance Imaging (MRI) of the brain and abdomen indicated a small anterior pituitary gland and liver masses, respectively. Liver biopsy showed steatohepatitis with bridging fibrosis (Figure 2). With GH therapy, her liver transaminases trended to normalcy (Table 1). Repeat MRI abdomen at 20 months after the start of GH showed stability of the liver lesions when compared to that done at 14 months. These hyperintense lesions like the initial ones were located in the liver's parenchyma and the appearance of the liver was otherwise normal.

**Figure 3:**
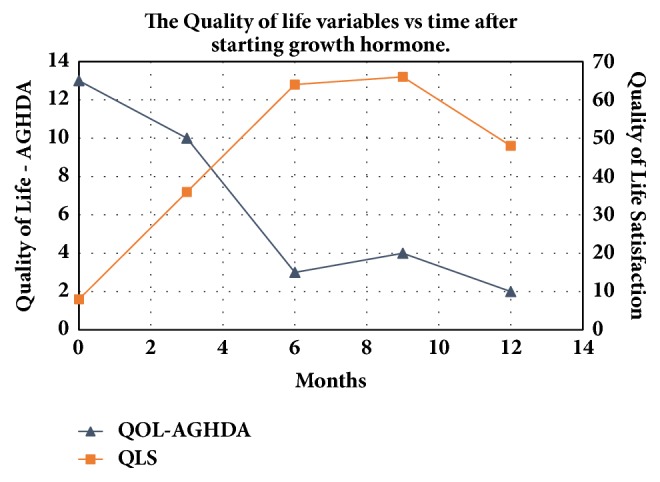
The patient's Quality of Life (QoL) scores with time. QoL-AGHDA: Quality of Life-Assessment of Growth Hormone Deficiency in Adults. QLS: Quality of Life Satisfaction. _1_A decrease in score for QoL-AGHDA indicates an improvement in QoL-AGHDA, whereas an increase in QLS indicates an improvement while taking GH therapy.

With GH therapy, the patient's stamina improved. She was now able to work for 20 hours weekly without becoming fully exhausted and her Quality of Life-Assessment of Growth Hormone Deficiency in Adults (QoL-AGHDA) and Quality of Life Satisfaction (QLS) scores, both questionnaire-based, improved (Figure 3).

## 3. Discussion

This case demonstrates that GH supplementation in an adolescent with CO-GHD led to improvements in transaminases, insulin requirements, and glucose control.

Several mouse models have corroborated the association of GHD with IR. In mice with liver GH receptor (GH-R) knockout, metabolic syndrome (MetS), steatohepatitis, increased inflammation, liver fibrosis, and hepatic tumor develop [4]. Additionally, a similar mouse model resulted in hyperinsulinemia, hyperglycemia, and IR. With the restoration of the liver's IGF-1 expression, there was an improvement in both insulin sensitivity and serum lipid profile. This, however, did not protect against hepatic inflammation induced by steatosis. This shows that GH and not IGF-1 directly affects lipid uptake and lipogenesis [5]. Also in a prior study again involving a liver specific GH receptor knockout mice, de novo lipogenesis was increased; however, this increase was not associated with the classic insulin mediated pathway [6]. So, our patient's IR and hepatic steatosis can be explained by her GHD based on data from some studies as well as the effect of GH in inhibiting this.

Evidence for the association of hepatic steatosis with GHD is also provided by the abnormalities involving the downstream pathway of GH signal transduction. Mice with hepatocyte-specific deletion of Janus kinase 2 (JAK2L), involved in the postreceptor phase of GH signaling, were lean but had FL. They also showed increased levels of GH, triglycerides, and plasma free fatty acids. Since GH in some instances can cause lipolysis, GH-deficient* little *mice which were crossed to JAK2L mice had both a rescuing of the FL and an increased expression of a fatty acid transporter [7]. Though this provides a mechanism for the FL observed with the liver specific disruption of GH signaling, in the same mice, elevated GH levels occurring as a consequence of disrupted signaling can cause an increase in resting energy expenditure [8]. In this situation, steatohepatitis is prevented based on increased fatty acid utilization. So the putative lipolytic action of GH can be offset by hyperinsulinemia; hence, the action of GH is variable and depends on the physiological context.

Additionally, mice with signal transducer and activator of transcription (STAT) 5 mutations, another downstream signal from GH, also develop steatohepatitis [9]. Since pathologies involving the downstream pathways in GH signal transduction are associated with steatohepatitis, this supports the role of GH in lipid metabolism and the notion that the FL changes in our patient may be due to GHD.

Clinical reports about Laron syndrome, primary GH insensitivity involving a molecular defect in human GH-R, have also documented the development of FL, IR, and T2DM [10]. In addition, men with hypopituitarism have a high prevalence of nonalcoholic FL disease (NAFLD) in the absence of GH therapy. When these men were treated with GH, there was histological improvement in the liver. This demonstrates that NAFLD is predominantly attributable to GH [11].

Though a follow-up liver biopsy was not done, the trend to normal in our patient's transaminases (Table 1) supports the effect of GH on the liver and the likelihood that these changes were induced by GHD. Since our patient's steatohepatitis improved with GH treatment, she likely had a reduction in her hepatic IR. The evidence for this was seen in her decreased insulin requirements. This reduction in IR also was associated with her decreased blood pressures and these decreased pressures can be explained since IR negatively impacts endothelial cell function [12].

Moreover, the decrease in our patient's insulin resistance may also be explained by the positive impact of GH on *β*-cell function which is well described. In adults with lifetime congenital untreated GHD, there is reduced *β*-cell function [13]. In children with GHD who are supplemented with GH, *β*-cell secretory capacity is enhanced [14]. Additionally, studies in mice have shown that, with isolated GHD, *β*-cell function deteriorates. This deterioration is not due to changes in *β*-cell mass [15]. Studies by* Nielsen* et al. have shown that GH stimulates *β*-cell proliferation, glucose induced insulin release, and insulin gene expression* in vitro *[16]. These also provide a possible mechanism for the decrease in our patient's IR.

Atorvastatin was added to our patient's treatment because although an amelioration of her LDL-C was expected with GH therapy, this did not happen. Plausible explanations for this include a genetic basis for her hyperlipidemia. This could have been superimposed on an accumulated adverse CV risk profile which developed based on the time period of her untreated CO-GHD [17]. Atorvastatin was also added to her treatment as well since based on her history of T2DM, her LDL-C levels needed improvement.

Based on the points discussed, with evidence from adult studies, clinical reports, and, more recently, mouse models (even with the lack of pediatric studies, especially long-term ones) explaining the important contribution of GH to normal lipid and glucose metabolism, there is enough data to reinforce the benefits of treating GHD even when linear growth is completed.

Pediatric clinicians should highlight and reinforce that GH supplementation has the potential to prevent adverse metabolic consequences in untreated states of severe deficiency. Also important is the fact that, even with growth cessation, supplementation with GH may improve QoL-AGHDA and QLS scores [18]. These questionnaire-based scores address the impact of GHD on issues of relevance to patients with GHD and can be useful for tracking the patient's response to treatment. The QoL-AGHDA tool addresses the general impact of GHD on each patient whereas the QLS score accounts for the level of importance which each individual may place on the issues affecting his/her life and gives a summarized weighted score based on these.

In conclusion, this case illustrates not only that both NAFLD and T2DM are potential associations of untreated GHD but also that they may represent points along the natural history of hepatic IR secondary to untreated GHD. Furthermore, clinicians should ensure that patients with CO-GHD are not only treated in childhood but also appropriately transitioned to adult GH dosing after growth has ceased. This is important as GH can have a crucial role beyond the period of linear growth. With delayed GH initiation, it is possible that, with irreversible hepatic injury (such as bridging fibrosis in this patient), there may not be total amelioration of the metabolic manifestations seen in patients with GHD.

## Figures and Tables

**Table 1:** Some of the patient's lab tests with reference to growth hormone start.

TEST	RESULT			REFERENCE RANGE
	**Baseline**	**12 mths**	**22 mths**	
**Aspartate aminotransferase**	92	36	33	< 40 U/L

**Alanine aminotransferase**	87	24	56	15- 50 U//L

**Total Cholesterol **	247	99	132	95- 195 mg/dL

**HDL Cholesterol **	32	27	43	40- 58 ng/dL

**LDL Cholesterol**	171	57	65	73- 117 mg/dL

**Triglyceride**	267	70	120	20-200 mg/dL

**IGF-1**	74	146	294	121-566 ng/mL

## Additional Points

*Learning Points.* (i) Growth hormone is integral to both glucose and lipid metabolism even after the period of linear growth. (ii) Insulin resistance (IR) can be a feature of untreated growth hormone deficiency (GHD). (iii) Hepatic steatosis and type 2 diabetes mellitus (T2DM) being both hepatic IR states could be a feature of the natural history of untreated GHD. (iv) While growth hormone supplementation may ameliorate IR seen in the setting of T2DM and GHD, it is likely that delayed supplementation may not fully ameliorate IR if simple steatosis progresses to steatohepatitis with fibrosis.
